# Characteristics of *Aspergillus fumigatus* in Association with *Stenotrophomonas maltophilia* in an *In Vitro* Model of Mixed Biofilm

**DOI:** 10.1371/journal.pone.0166325

**Published:** 2016-11-21

**Authors:** Elise Melloul, Stéphanie Luiggi, Leslie Anaïs, Pascal Arné, Jean-Marc Costa, Vincent Fihman, Benoit Briard, Eric Dannaoui, Jacques Guillot, Jean-Winoc Decousser, Anne Beauvais, Françoise Botterel

**Affiliations:** 1 EA 7380 Dynamyc, Université Paris Est Créteil, Ecole nationale vétérinaire de d’Alfort, IMRB, Créteil, France; 2 Ecole nationale vétérinaire de Maisons-Alfort, Maisons-Alfort, France; 3 Cerba, Saint-Ouen l’Aumône, France; 4 Unité de Bactériologie-Hygiène, AP-HP, DHU VIC, Hôpital Henri Mondor, Département de Microbiologie, Créteil, France; 5 Unité des Aspergillus, Institut Pasteur, Paris, France; 6 Unité de Parasitologie—Mycologie, Département de Microbiologie, Hôpital Européen Georges Pompidou, Paris, France; 7 Unité de Mycologie, Département de Microbiologie, Groupe hospitalier Henri Mondor—Albert Chenevier, APHP, DHU VIC Université Paris-Est- Créteil, Créteil, France; J Craig Venter Institute, UNITED STATES

## Abstract

**Background:**

Biofilms are communal structures of microorganisms that have long been associated with a variety of persistent infections poorly responding to conventional antibiotic or antifungal therapy. *Aspergillus fumigatus* fungus and *Stenotrophomonas maltophilia* bacteria are examples of the microorganisms that can coexist to form a biofilm especially in the respiratory tract of immunocompromised patients or cystic fibrosis patients. The aim of the present study was to develop and assess an *in vitro* model of a mixed biofilm associating *S*. *maltophilia* and *A*. *fumigatus* by using analytical and quantitative approaches.

**Materials and Methods:**

An *A*. *fumigatus* strain (ATCC 13073) expressing a Green Fluorescent Protein (GFP) and an *S*. *maltophilia* strain (ATCC 13637) were used. Fungal and bacterial inocula (10^5^ conidia/mL and 10^6^ cells/mL, respectively) were simultaneously deposited to initiate the development of an *in vitro* mixed biofilm on polystyrene supports at 37°C for 24 h. The structure of the biofilm was analysed via qualitative microscopic techniques like scanning electron and transmission electron microscopy, and fluorescence microscopy, and by quantitative techniques including qPCR and crystal violet staining.

**Results:**

Analytic methods revealed typical structures of biofilm with production of an extracellular matrix (ECM) enclosing fungal hyphae and bacteria. Quantitative methods showed a decrease of *A*. *fumigatus* growth and ECM production in the mixed biofilm with antibiosis effect of the bacteria on the fungi seen as abortive hyphae, limited hyphal growth, fewer conidia, and thicker fungal cell walls.

**Conclusion:**

For the first time, a mixed *A*. *fumigatus*—*S*. *maltophilia* biofilm was validated by various analytical and quantitative approaches and the bacterial antibiosis effect on the fungus was demonstrated. The mixed biofilm model is an interesting experimentation field to evaluate efficiency of antimicrobial agents and to analyse the interactions between the biofilm and the airways epithelium.

## Introduction

Biofilm is composed of densely packed colonies of microorganisms enclosed in a matrix of self-produced extracellular polymeric substance, and adhered to an abiotic or biotic surface. The microcolonies forming the biofilm are either mono- or multi-species of mono- or multi-kingdom populations (e.g. bacterial, fungal and mixed biofilms).

Biofilms represent a protective environment enabling microorganisms to thrive in a hostile surrounding, and show inherent resistance to antimicrobial agents. Both factors give rise to many persistent and chronic infections including chronic middle ear infection, chronic sinusitis or otitis, and chronic lung disease [1]. Recent studies have described *Aspergillus fumigatus* colonization in patients with chronic lung diseases, especially chronic obstructive pulmonary disease (COPD) and cystic fibrosis, and suggested a probable link with lung structural changes and heavy antibiotic courses [2, 3].

Among emerging microorganisms concomitantly isolated with *A*. *fumigatus* from the respiratory tract of immunocompromised patients or those suffering from chronic respiratory diseases, *Pseudomonas aeruginosa*, a non-fermentative Gram-negative bacillus, is the most studied [4]. Other bacteria, such as *Stenotrophomonas maltophilia*, have recently been identified as an important hospital-associated pathogen colonising the same population of patients. This intrinsically multidrug-resistant, saprophytic, and ubiquitous microorganism belongs to the "*Pseudomonas* and parented" group of bacteria and is increasingly encountered in human infectious diseases [5, 6]. Though intrinsically not highly virulent, its environmental dissemination and resistance to selective pressure antibiotics promote its opportunistic pathogenicity in immunocompromised patients. The risk factors associated with *S*. *maltophilia* colonization and/or infections are often shared with *Aspergillus* infections, and include immunosuppressive or corticosteroid therapy and a history of long-term intake of broad-spectrum antibiotics [7].

Fungi and bacteria are often simultaneously cultivated from respiratory tract specimens but the physiopathological, biological, and clinical relevance of the microbial association remains controversial [8–10]. In cystic fibrosis (CF), significant associations have been reported between the upper airways colonization by *A*. *fumigatus*, and the presence of either *S*. *maltophilia* [11, 12], or *P*. *aeruginosa* or atypical mycobacteria [12, 13]. Similarly, bronchial colonization by *S*. *maltophilia* is independently associated with the development of allergic bronchopulmonary aspergillosis (ABPA) [14].

Intrinsic interactions between fungi and bacteria have favoured their co-evolution, and initiated polymicrobial mechanisms of synergism, antagonism, and mutualism. For instance, *P*. *aeruginosa* exhibit antibiosis to *A*. *fumigatus* by direct contact and secreted extracellular molecules [15, 16]. Microscopic observations of bacteria-fungi interactions showed that at least some antagonistic bacteria actively move towards fungal hyphae and colonize their surface [17]. *In vitro*, biofilms with single *A*. *fumigatus* [18–21] or *S*. *maltophilia* [22–24] showed standard growth, yet and to our knowledge, no study on mixed biofilm of these two organisms has been conducted. We hypothesised that, based on these clinical and fundamental findings, *S*. *maltophilia* and *A*. *fumigatus* might interact in CF lung, especially in the form of a biofilm. The objectives of the present study were to develop an *in vitro* model of *A*. *fumigatus* and *S*. *maltophilia* biofilm by using both analytical (scanning electron SEM, transmission electron microscopy TEM, and fluorescence microscopy) and quantitative (crystal violet staining and qPCR) approaches.

## Materials and Methods

### Microbial strains and growth conditions

*Aspergillus fumigatus* strain (ATCC 13073) from glycerol stocks stored at -20°C was streaked out on fresh 2% malt agar slants with 0.05% of chloramphenicol for 7 days at 37°C. This modified strain, which express GFP, was provided by J.A. Wasylnka [25]. Conidia were harvested after 7 days at 37°C by rinsing the slants with phosphate-buffered saline (PBS) supplemented with 0.1% Tween 20 (PBST). Conidia were filtered through a 40-μm pore-size cell strainer (Millipore, Molsheim, France) to remove the mycelium.

The strain of *Stenotrophomas maltophilia* (ATCC 13637) is a clinical isolate which was stocked at -20°C in glycerol and streaked out on fresh CHO-plate (Columbia agar + 5% horse blood) (BioMérieux, Marcy-l'Etoile, France) for 24 h at 37°C.

### Biofilm formation

#### Preparation of *A*. *fumigatus* inoculum

*Aspergillus fumigatus* conidia were transferred into PBST and centrifuged for 10 min at 2500 rpm. The conidia were suspended in 1 mL of PBS and counted using Malassez counting chamber to adjust to a final concentration of 10^5^ conidia/mL. This concentration was obtained by serial dilution in 3 (N-morpholino)—propanesulphonic acid (MOPS)—buffered RPMI 1640 [pH 7.0] (Sigma-Aldrich, France) to which was added 10% of FBS (foetal bovine serum) (Sigma-Aldrich, France). Serum was added to the biofilm media as the main carbon source because it is present *in vivo* and promotes the growth of a hyphal network, the main component of fungal biofilms.

#### Preparation *S*. *maltophilia* inoculum

*Stenotrophomonas maltophilia* colonies were collected and diluted in brain heart infusion medium (BHI) (BioMérieux, Marcy-l'Etoile, France). Strains were grown overnight in an orbital shaker at 250 rpm. The suspension was adjusted to opacity of 0.5 MacFarland (≈1x10^8^ cells/mL). A 1:100 dilution was made in MOPS-buffered RPMI 1640 [pH7.0] supplemented with 10% FBS to obtain a working concentration of 10^6^ cells/mL.

#### Biofilm formation

The first set of experiments included the preparation of single *A*. *fumigatus* or *S*. *maltophilia*-containing biofilms which were then validated before the preparation of mixed biofilms (*A*. *fumigatus* and *S*. *maltophilia*). Single and mixed biofilms were compared between each other in all experiments.

The single and mixed biofilms were produced on polystyrene supports, in 96-well microtiter plates (Thermo Fisher Scientific Inc, France) for quantitative analysis or on Lab-Tek^TM^ slides (Nunc^TM^, Thermo Fisher Scientific Inc, France) for microscopic analyses.

For the single biofilms, 100 μL of the microbial suspension (bacteria or fungal conidia) were inoculated in 100 μL of MOPS-buffered RPMI + 10% FBS. For the mixed biofilms, 100 μL of the fungal conidia solution and 100 μL of the bacterial solution were simultaneously added. The microbial concentrations, used for single and mixed biofilm formation, were 10^5^ conidia/mL for *A*. *fumigatus* and 10^6^ cells/mL for *S*. *maltophilia*, as previously described in the studies of Mowat et al. [16, 26].

Cultures were incubated at 37°C in static condition between 8 and 24 h for microscopic biofilm analyses. Kinetics of biofilm formation was performed between 0 and 24 h for quantitative and qualitative analyses. To eliminate planktonic cells, cultures were washed twice with PBS.

### Microscopic analysis

Confocal laser scanning microscopy (CLSM) was used to analyse the kinetics of formation of the single fungal and mixed biofilms, the phenotype of *A*. *fumigatus*, and the biofilm thickness. Electron microscopies were used to analyse the extracellular matrix (ECM) formation, the bacteria-fungi interactions, and the thicknesses of fungal cell wall. All the experiments have been performed in duplicate.

#### Scanning electron microscopy (SEM)

After the biofilm production, cultures destined for SEM were air-dried and fixed with a solution of 2.5% glutaraldehyde at 4°C for 4 h and then dehydrated in a series of aqueous ethanol solutions (70 to 100%). Samples were transferred on a support and metallised with gold by a JEOL JFC-1100E Ion Sputtering device. The single *S*. *maltophilia* and *A*. *fumigatus* biofilms, and the mixed biofilm were finally examined using a JEOL JSM-6301F FESEM instrument (Japan).

#### Transmission electron microscopy (TEM)

Biofilm samples for TEM were first incubated 10 min at 4°C and then fixed with a solution of 2.5% glutaraldehyde diluted in sodium cacodylate buffer (pH 6.5) for 3 min. The solution was removed and then a similar volume of 2.5% glutaraldehyde solution was added and the whole mix was incubated at 4°C for 20 min. The cultures were finally placed at 4°C in sodium cacodylate buffer (pH 6.5). The biofilm samples were then post-fixed with osmium tetroxide and dehydrated with different dilutions of alcohol (50–100%). The samples were then embedded into EPON resin and left to polymerize. Ultra-fine sections were obtained using a Leica UC7-RT ultramicrotome, and contrasted with lead-citrate and uranyl-acetate solutions. Finally, specimens were mounted on grids to be examined under the microscope (JEOL 100 CX II instrument, Japan). TEM was used to measure the cell wall thickness of *A*. *fumigatus* in single and mixed biofilms using the ImageJ program (http://imagej.nih.gov/ij/). For both biofilms, between 10 and 20 measurements of cell wall thickness were performed on 27 hyphae.

#### Confocal laser scanning microscopy (CLSM)

Investigations of biofilm formation and phenotypic modifications of fungi in the presence of the bacteria were carried out by CLSM. Single *A*. *fumigatus* biofilm and mixed biofilm (*A*. *fumigatus* + *S*. *maltophilia*) were produced and their formation was arrested 8, 12, 16 or 24 h after inoculation by PBS washes. Concanavalin A (ConA) coupled with tetramethylrhodamin (25 μg/ml) (Invitrogen, France) were used to visualize the extracellular matrix (ECM) with TRITC/A546 filter, in accordance with the adapted method of Shopova *et al*. [27]. After adding ConA, the samples were incubated at 37°C for 45 min at 250 rpm, and then biofilms were washed 3 times with PBS. GFP strain of *A*. *fumigatus* was visualised with FITC/A488 filter. The biofilms were examined under Zeiss LSM 510 META microscope (Zeiss, Germany). CLSM was used to measure biofilm thickness using the ImageJ program. For single and mixed biofilms, the measurements were performed on three different samples of each. On the whole, 50 measurements taken at different locations of the single and mixed biofilms were analysed.

### Assessment of biomass by crystal violet method

To evaluate the biomass formation, crystal violet staining method was applied on single and mixed biofilms every 4 hours (from 8 to 24 h of growth after inoculation). This experiment was repeated three times using eight wells per biofilms each time. Biofilms were produced in 96-well plates and then washed with PBS. Afterward, 250 μL of crystal violet (0.01%) was added to each well and the samples were incubated for 30 min at room temperature and washed two times with PBS before adding acetic acid (30%). The inspection was performed on the spectrophotometer at 550 nm (Multiskan^TM^ FC, Thermo Fisher Scientific Inc., France). The optical density (OD) values are proportional to the quantity of biofilm biomass. Absorbance results of control (RPMI) were subtracted from the single and mixed biofilms results. The adjusted values of absorbance were used to compare the growth of the single with that of the mixed biofilms. The biomass of the mixed biofilm was expressed as a percentage of the ratio: mixed biofilm OD / single *A*. *fumigatus* biofilm OD.

### Assessment of conidial and bacteria equivalent by real time PCR

The biofilms were washed twice before incubation for 1 h at 56°C with 250 μL of Magna Pure bacteria lysis buffer (Roche, Meylan, France). They were then scraped to recover all adherent organisms, and washed with 300 μL PBS. The samples were then homogenized as previously described [28] and extracted with the QIAamp DNA Mini Kit (Qiagen, Courtaboeuf, France) according to the manufacturer's instructions.

Quantification of the amount of *A*. *fumigatus* and *S*. *maltophilia* DNA was performed by qPCR targeting the 28S rRNA and 23S rRNA genes respectively. The 28S primers and probes sequences were reported by Challier et al. [29]. The sequences of 23S were: 23S-270 (5’-CTG GAT TGG TTC TAG GAA AAC GC-3’) and 23S-339 (5’-CTA CTC GTC TTC ACT GGA ATG GC-3’), and the hybridization 23S probe (5’-VIC-GAG CGG CCA TAG AAG GTG ATA GCC CTG-TAMRA-3’). Each DNA sample was treated in duplicate by using the LightCycler^®^ 480 apparatus (Roche Diagnostics, Meylan, France). The reaction mixture consisted in 5 μL of DNA, 400 nM sense and antisense primers from 28S or 23S targets, 200 nM 28S probe or 23S probe, and LightCycler^®^ 480 Probes Master with a final concentration of 1X (Roche, Meylan, France). All assays were run under the following conditions: 95°C for 10 min, then 50 cycles of 15 s at 95°C and 1 min at 60°C.

Fluorescence curves were analysed using LightCycler software V3.5 and results were expressed in conidial equivalent (CE) or bacterial equivalent (BE) in comparison with a standard curve blotted on DNA samples extracted from co-inoculated solutions with different doses of *A*. *fumigatus* conidia (1 to 10^8^ conidia) and *S*. *maltophilia* (10 to 10^9^ cells).

DNA quantification of bacteria or fungi was done on the single and mixed biofilms every 4 hours between 0 and 24 h to evaluate growth of microorganisms in both situations. This experiment was performed in triplicate using three samples per biofilm type and per time.

### Statistical analysis

Statistical analyses were performed using JMP 12.0 software. Data of continuous variables are presented in means ± standard errors of the mean. P-value was significant if <0.05. Shapiro-Wilk test was used to examine normality of the data, and the results showed that all variables did not have normal distribution, hence the use of non-parametric tests.

Data were analysed by Kruskal-Wallis (one-way analysis of variance) test in order to compare the percent of biomass at different times at which biofilm production was arrested. Linear regression was applied to assess the variation of OD over time for the bacterial, the fungal, and the mixed biofilms. The quantity of DNA of *S*. *maltophilia* and *A*. *fumigatus* in the single or mixed biofilms over time was also analysed with linear regression. Mann-Whitney U test was used to compare the DNA result (LogCE/mL) of *A*. *fumigatus* of the single biofilm with that of the mixed biofilms at T = 12 h. Microscopy data (TEM and CLSM) were subjected to Wilcoxon and Kruskal-Wallis tests in order to compare the means of fungal cell wall thickness in the single and mixed biofilms, and at different times or biofilms thicknesses.

## Results

### Characteristics of the single and mixed biofilms

#### Kinetics of biofilms formation

**Fig 1:**
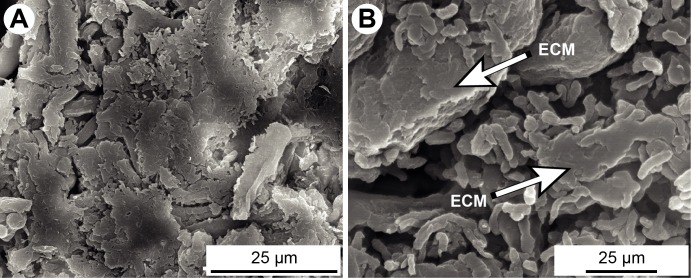
Formation of *in vitro* biofilm of *S*. *maltophilia* observed by SEM. (**A-B**) Single biofilm of *S*. *maltophilia* after 24 h of culture. ECM = extracellular matrix.

**Fig 2:**
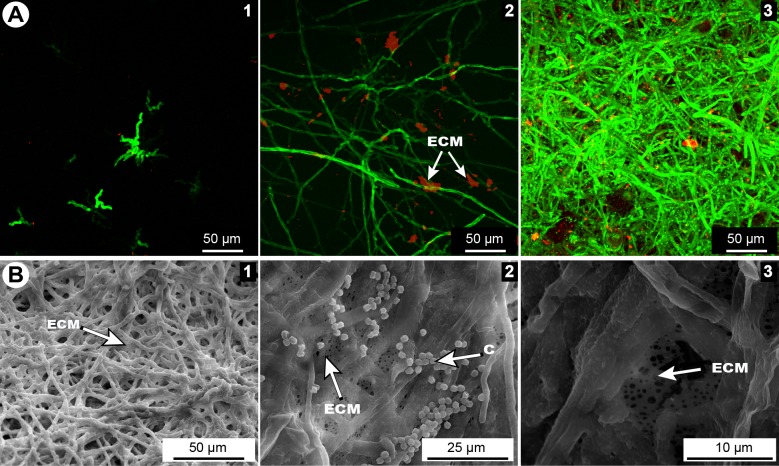
Formation of *in vitro* biofilm of *A*. *fumigatus*. (**A**) Kinetics of biofilm formation visualized in CLSM: 8 h (1), 12 h (2) and 24 h (3) after inoculation. (**B**) 24 h–old biofilm in SEM: general aspect of Af biofilm (1), hyphae embedded in ECM and presence of conidia (2), ECM with holes (3). ECM = extracellular matrix, C = conidia.

The inoculation of *S*. *maltophilia* and *A*. *fumigatus* on polystyrene supports at 37°C in static condition was essential for the development of biofilms. SEM analysis of the single bacterial cultures sections showed typical rod shape Gram-negative bacilli (Fig 1A). The bacteria were embedded in the ECM, as expected upon biofilm formation (Fig 1B). The kinetics of formation of *A*. *fumigatus* biofilm was observed by CLSM (Fig 2A). After 8 h of culture, the conidia adhered to the surface and germinated to form young hyphae. After 12 h of culture, all conidia germinated, and the yielded young hyphae matured to form a dense network. At this time, the ECM composed of mannose and glucose residues and coloured in red by ConA marker, was produced (Fig 2A2). At 24 h of growth, a highly branched mycelium was observed and the production of ECM increased. SEM analyses of the 24 h-old biofilm showed hyphae totally embedded in the ECM (Fig 2B1), which in turn appeared thick with scattered holes (Fig 2B2 and 2B3). Conidial heads and conidia were also observed in the fungal biofilm (Fig 2B2).

**Fig 3:**
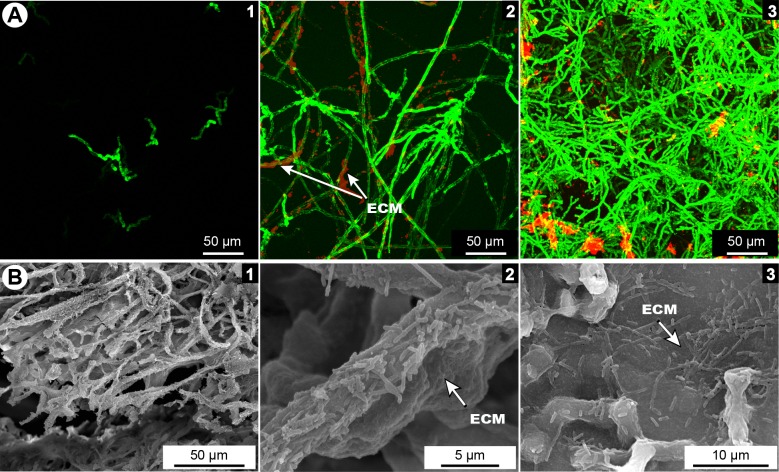
Formation of *in vitro* mixed biofilm of *S*. *maltophilia* and *A*. *fumigatus*. (**A**) Kinetics of mixed biofilm formation visualized in CLSM: 8 h (1), 12 h (2) and 24 h (3) after inoculation. (**B**) 24 h–old biofilm in SEM: general aspect of mixed biofilm (1), bacteria covering *A*. *fumigatus* hyphae and embedded in ECM (2), bacteria between hyphae and embedded in ECM (3). ECM = extracellular matrix.

In the co-culture samples, the two microorganisms and the ECM were visualised by optic microscope and SEM like in the single biofilms, proving that a mixed biofilm of *A*. *fumigatus* and *S*. *maltophilia* was generated (Fig 3). As for the *A*. *fumigatus* biofilm, the conidia germinated after 8 h of culture, and the mycelium started to grow at 12 h (Fig 3A1 and 3A2). At the same time, the ECM was produced and was seen surrounding the hyphae. After 24 h, the hyphae formed a 3D structure and the ECM volume increased (Fig 3A3) which indicates that the biofilm reached a phase of maturation. Several aggregates of bacteria were detected by SEM analyses on the surface of the hyphae (Fig 3B1 and 3B2) and between them (Fig 3B3). In both cases, the bacteria were embedded in the ECM.

#### Phenotype modifications of *A*. *fumigatus* in the presence of *S*. *maltophilia*

**Fig 4:**
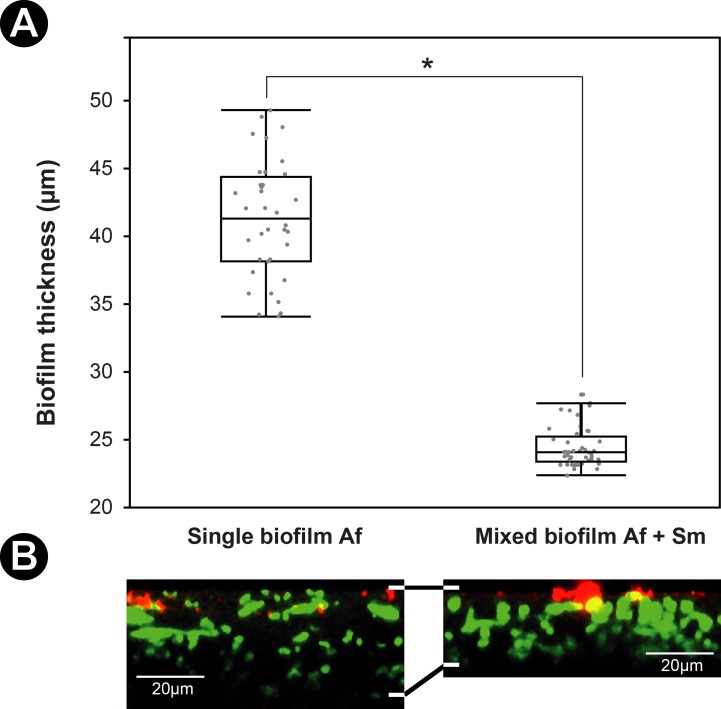
*A*. *fumigatus* and mixed biofilms thicknesses. (**A**) Means of *A*. *fumigatus* and mixed biofilms thicknesses after 24 h of culture (**B**) CLSM observations of 24 h-old biofilms thicknesses inoculated on Lab-Tek^TM^ slides. Sm = *S*. *maltophilia*, Af = *A*. *fumigatus*. For each biofilm, 50 measurements were taken. Results are expressed in mean±SE, * *p* < 0.0001.

**Fig 5:**
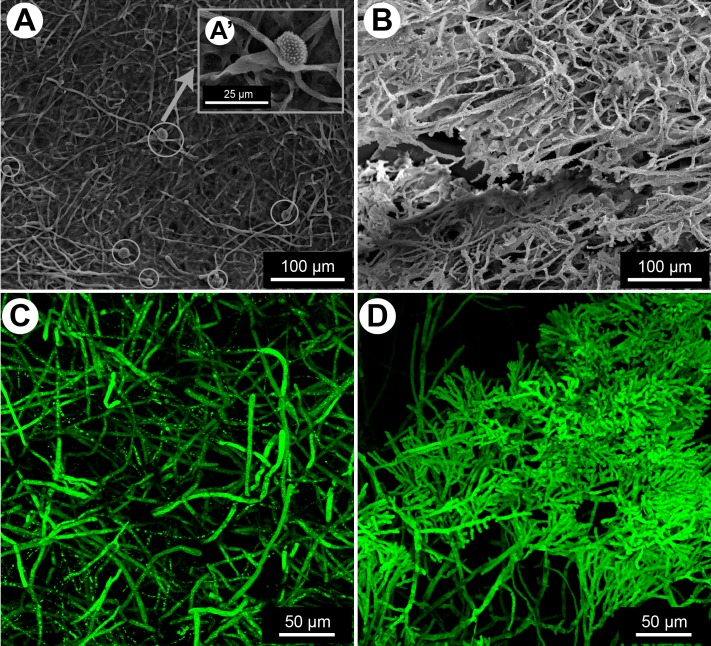
**Conidiation and phenotype of *A*. *fumigatus* in the mixed biofilm visualized on SEM (A, B) and CLSM (C, D).** (**A, C**) 24 h-old single *A*. *fumigatus* biofilm (A’) zoom on the presence of conidial head (**B, D**) 24 h-old mixed biofilm of *A*. *fumigatus* and *S*. *maltophilia*. Grey circle represents conidial head of *A. fumigatus* which is only present in the single biofilm.

**Fig 6:**
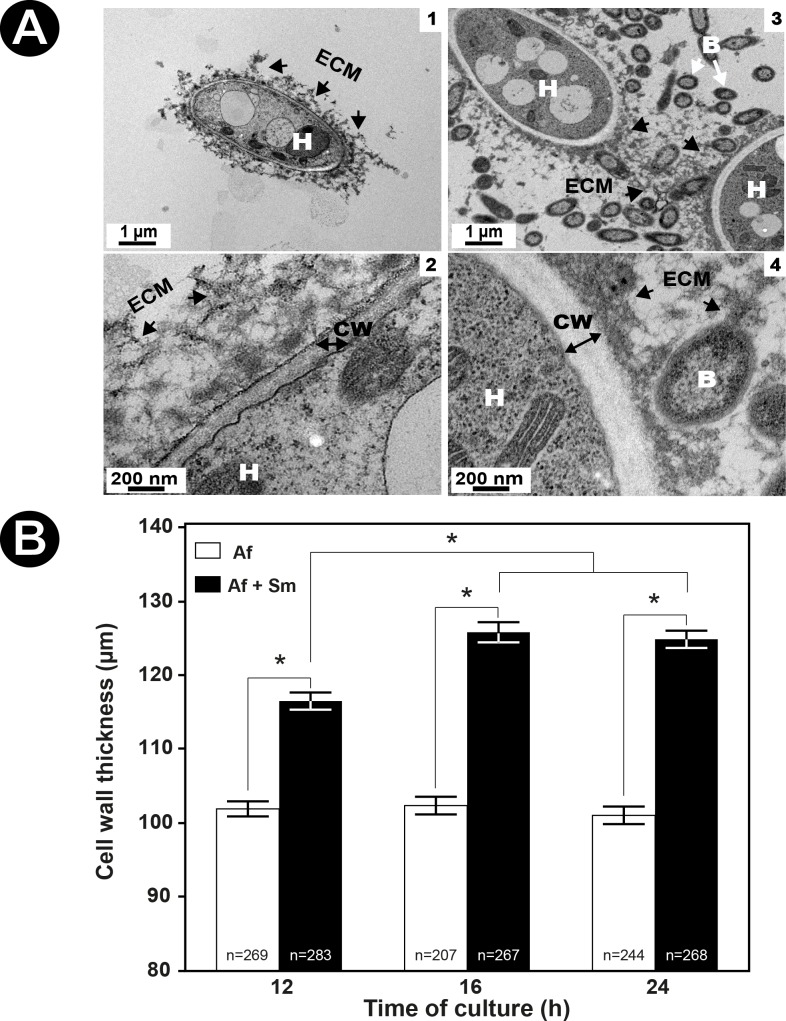
Cell wall thickness of *A*. *fumigatus* in the single and mixed biofilms. (**A**) Observation on 24 h–old single *A*. *fumigatus* biofilm (1–2) and mixed biofilm (3–4) by TEM (**B**) Cell wall thickness of *A*. *fumigatus* measured on TEM images of the single and mixed biofilms. H = hyphae, B = bacteria, CW = cell wall, ECM = extracellular matrix, Sm = *S*. *maltophilia*, Af = *A*. *fumigatus*. For each biofilm, approximately 15 measurements on 27 hyphae were taken. Results are expressed in mean±SE, * *p* < 0.0001.

Differences between the single and mixed biofilms were subsequently observed after 24 h of culture i.e. during the maturation phase of the fungal biofilm where both microorganisms were completely embedded in the ECM. Furthermore, *A*. *fumigatus* started asexual reproduction at 24 h of culture. When *A*. *fumigatus* is in the biofilm with *S*. *maltophilia* for 24 h, the fungal growth and the morphological aspects of the hyphae were both modified compared with the single *A*. *fumigatus* biofilm. The CLSM analysis of the biofilms allowed us to measure and to compare the means of thickness of the fungal and mixed biofilms that were respectively 41.3±4.3 μm and 24.4±1.5 μm (Fig 4A). When the fungus was in contact with the bacteria, the growth of its hyphae was delayed, and the thickness of the mixed biofilm was found significantly less than that of the fungal biofilm (p<0.0001) (Fig 4B). The hyphal network appeared less dense in the mixed biofilm than in the single fungal biofilm and no conidiation of *A*. *fumigatus* was observed at 24 h in the mixed biofilm compared with the single fungal biofilm (Fig 5A and 5B). Moreover, atypical hyphae exhibiting atrophied structures and highly branched patterns with shorter ramifications at the tip were seen in the mixed biofilm (Fig 5C and 5D). The TEM observations of the single and mixed biofilm showed that the hyphae were enclosed by the ECM (Fig 6A). In the mixed biofilm, the ECM covered the fungal cell wall and the bacteria adhered to the surface of the hyphae (Fig 6A2). TEM also revealed that there was a direct interaction between the bacteria and the fungus via the ECM (Fig 6A4). The bacteria were encapsulated in extracellular materials on the surface of the fungal cell wall (Fig 6A4). Furthermore, the results of TEM confirmed that the cell wall of *A*. *fumigatus* in the mixed biofilm, in the presence of the bacteria, was significantly thicker than it was in the single biofilm (Fig 6A2 and 6A4) at 12, 16, and 24 h of culture (p<0.0001) (Fig 6B). In the single biofilm of *A*. *fumigatus*, the means of fungal cell wall thicknesses at the same time-points were respectively 101.9±1.0 nm, 102.4±1.2 nm, and 102.4±1.2 nm and were not significantly different (p = 0.4768). In contrast, the means of fungal cell wall thicknesses in the mixed biofilms for the same three time-points (12, 16, and 24 h) were respectively 116.5±1.2 nm, 125.8±1.4 nm, and 124.8±1.2 nm. This indicates that the cell wall thickness of *A*. *fumigatus* increased before 12 h and continued until 16 h of culture when the fungus was in contact with *S*. *maltophilia* (p<0.0001), and then stopped. There was no difference between the means of cell wall thicknesses at 16 and 24 h (p = 0.8643) (Fig 6B).

### Quantification of the single and mixed biofilms

#### Biomass quantification

**Fig 7:**
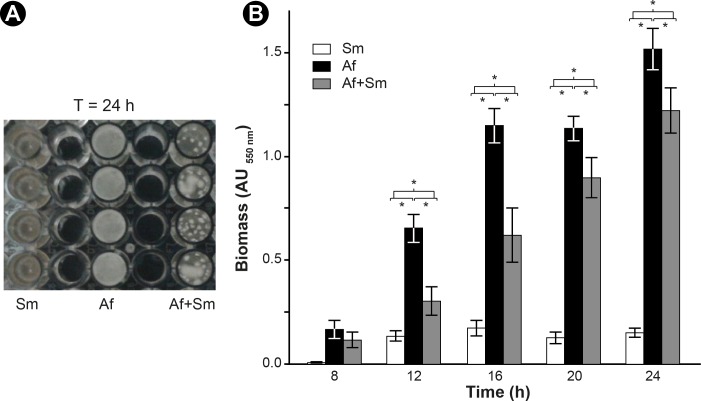
Quantification of biofilms biomasses by crystal violet staining. (**A**) Macroscopic observations of biofilms in 96-well plate after 24 h of culture (**B**) Biomasses of the single and mixed biofilms are measured by optical density as a function with respect to time. Single biofilms: Af = *A*. *fumigatus* biofilm, Sm = *S*. *maltophilia* biofilm, Af+Sm = mixed biofilm. The experiment was repeated 3 times, using 8 wells per biofilm. Results are expressed as by the mean±SE, * *p* < 0.05.

Crystal violet is traditionally used to quantify the biomass (organisms and ECM) of a biofilm. As expected, the *S*. *maltophilia* biomass was marginal compared with that of *A*. *fumigatus* and of the mixed biofilms (p<0.0001) (Fig 7A and 7B). The *S*. *maltophilia* biomass increased between 8 and 16 h (p<0.0001), then its development slowed down after 16 h. The biofilm biomass was stable between 16 and 24 h (p = 0.0506). A significant increase of the *A*. *fumigatus* and the mixed biofilms biomasses was observed during the whole incubation period (p<0.0001), suggesting that the fungus was still in the growth phase until 24 h (Fig 7B). A slower biomass production was observed in the mixed biofilm compared with the single *A*. *fumigatus* biofilm. This difference was statistically significant between 12 and 24 h of incubation (p<0.005). The slowing in the growth of *A*. *fumigatus* in the mixed biofilm occurred during the growth phase of *S*. *maltophilia* with a reduction of the biomass by 54% between 12 and 16 h. A less remarkable decrease of 20% in the fungal biomass was observed as the bacterial biomass production slowed down between 20 and 24 h in the mixed biofilm (Fig 7B).

#### Quantification of fungal and bacterial microorganisms

**Fig 8:**
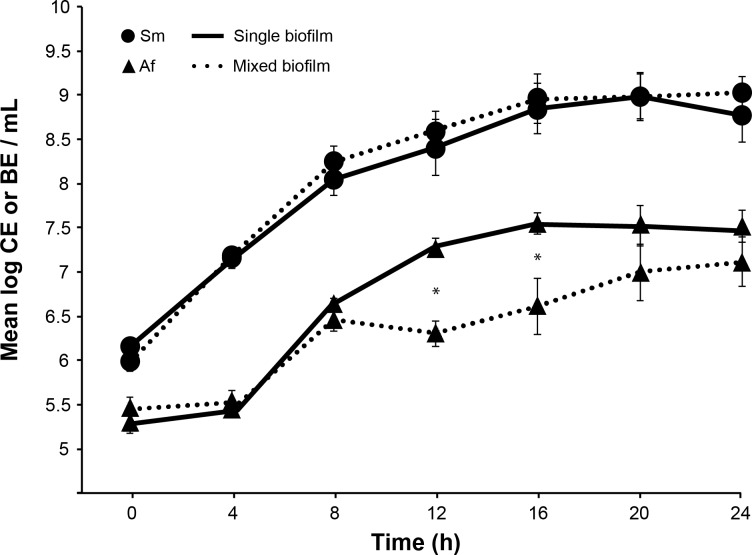
Growth of *S*. *maltophilia* and *A*. *fumigatus* in the single and mixed biofilms. Data are expressed in log of CE or BE/mL as measured by qPCR over 24h and presented in the form of mean±SE. Sm = *S*. *maltophilia*, Af = *A*. *fumigatus*. The experiment was repeated 3 times, using 3 wells per biofilm. Results are expressed in mean±SE, * p < 0.05 compared with the single biofilms.

Quantitative PCR was used to evaluate the growth of each microorganism in the mixed biofilm. As expected, we registered the means of 2.5x10^5^ and 3x10^5^ conidial equivalent/mL (CE/mL) for *A*. *fumigatus* and 1.5x10^6^ and 9.5x10^5^ bacterial equivalent/mL (BE/mL) for *S*. *maltophilia* at T0 in the single and mixed biofilms respectively. Our results perfectly match the initial concentrations we used for both microorganisms (Fig 8) as the bacteria were inoculated with 1log more than the fungi at T0.

Over the 24 h incubation period, a significant growth of *A*. *fumigatus* from 2.5x10^5^ to 3.4x10^7^ CE/mL (p<0.0001), and of *S*. *maltophilia* from 1.5x10^6^ to 6.3x10^8^ BE/mL (p<0.0001) within the single biofilms were observed. In the mixed biofilm, *A*. *fumigatus* grew from 3x10^5^ at T0 to 1.3x10^7^ CE/mL at T24 (p<0.0004), whereas *S*. *maltophilia* increased from 9.5x10^5^ to 1.2x10^9^ BE/mL (p<0.0001). The growth of *S*. *maltophilia* in the mixed biofilm was not significantly different from their growth in the single biofilm. Typical logistic growth of *S*. *maltophilia* was observed reaching a maximum of approximately 10^9^ BE/mL at 16 h (Fig 8).

For *A*. *fumigatus*, the growth was delayed compared with the bacteria and started after 4 h of incubation in the form of conidia swelling and initiation of germ tubes formation. Between 4 and 24 h of biofilm formation, the fungus passed through a growth phase consisting in hyphal development and biofilm production(Fig 8).

For the mixed-biofilm formation, the growth of *A*. *fumigatus* was hampered between 8 and 12 h of incubation, which was not the case in the single *A*. *fumigatus* biofilm (Fig 8).

In the single biofilm, the growth rate of *A*. *fumigatus* between 8 and 12 h was approximately 4x10^6^ CE/h, and the fungi continued to grow until 16 h with a mean growth rate of 4x10^6^ CE/h, and then stopped between 16 and 24 h. In contrast, in the mixed biofilm the growth rate between 8 and 12 h was equal to zero. Then, the fungi resumed their growth after 12 h and continued until 24 h with a mean rate of 10^6^ CE/h (Fig 8). Thus, the differences of conidial equivalent concentrations between the single and mixed biofilms were statistically significant at 12 h and 16 h time points (p = 0.0039), but not after (Fig 8).

## Discussion

*A*. *fumigatus* fungus is commonly present in the airways of CF or immunocompromised patients. It can interact with other microorganisms, such as *S*. *maltophilia* [12], one of the most common emerging multi-drug resistant organisms which thrive in the airways of the same patients [30]. These microorganisms can produce biofilms *in vitro* and *in vivo* [22–24, 31]. For such, further information on the interactions between *A*. *fumigatus* and *S*. *maltophilia* is of paramount importance. In the literature, some authors have studied interactions between *A*. *fumigatus* or *S*. *maltophilia* and other microorganisms [15, 32–35], but never between these two microorganisms. The originality of this study was to develop for the first time an *in vitro* model of mixed biofilm of *A*. *fumigatus* and *S*. *maltophilia* together, and to evaluate the effect of their interactions on the development of each.

To our knowledge, qPCR was previously used to quantify bacterial biofilms [36, 37] and *Candida* biofilm [38], but this is the first time it is used on a mixed biofilm of bacteria and filamentous fungi. However, the major drawback of this technique is the overestimation of cell numbers due to the presence of extracellular DNA and DNA originating from dead cells. The biomass of *S*. *maltophilia* biofilm increased until reaching a plateau at 16 h (Fig 7), which is similar to what was showed in the study of Di Bonaventura *et al*. [22]. The same was confirmed on qPCR; the bacteria in the single biofilm grew between 0 and 16 h then entered a stationary phase until 24 h (Fig 8). After 24 h of culture, the bacteria formed aggregates that were enclosed in a dense film of ECM (Fig 1), in consistency with what was previously described in other studies [22, 39]. In our study, the fungal biofilm formation was similar to the one described by Mowat *et al*. [26] as swollen conidia started germination between 4 and 8 h in order to form a monolayer of hyphae, followed by an intense filamentation to create a complex hyphal network at 24 h of culture (Fig 2A). The production of ECM in the fungal biofilm was visible at 12 h and increased until 24 h, the maturation phase, where the fungi was totally covered by the matrix (Fig 2), in consistency with the results of Beauvais and Latgé [18]. The microscopic analyses of this biofilm helped visualise the hyphae in the ECM which formed a resistant structure covering the hyphae and the spaces between them (Fig 2B) [21].

The simultaneous co-inoculation of *A*. *fumigatus* and *S*. *maltophilia* suspensions in static condition allowed us to develop a stably adherent mixed biofilm. The cells of *S*. *maltophilia* were in contact with *A*. *fumigatus* cell wall and embedded in the ECM (Fig 3). Most of the published studies on *in vitro* fungal and bacterial biofilms were performed with yeasts and bacteria [40–42], and very few models worked with filamentous fungi and bacteria, such as *A*. *fumigatus* and *P*. *aeruginosa* [16, 34] or *A*. *fumigatus* and *Staphylococcus aureus* [35]. An example of cellular structures that can support such interactions is cellulose, which is synthesized by several bacterial species and has a critical role in bacterial attachment to fungi, and in particular to chitin. The study of Brandl *et al*. indicates that cellulose–chitin interactions are required for the production of mixed *Salmonella* and *A*. *niger* biofilms. Other cellulose-producing bacterial pathogens may similarly interact with pathogenic *Aspergillus* species and thereby aggravate human illness [43]. In some cases bacteria provide fungi with compounds that enhance the production of fungal virulence determinants [10]. However, other bacteria produce factors that are likely to inhibit pathogenesis by repressing fungal filamentation, e.g. *P*. *aeruginosa* phenazines repress *C*. *albicans* filamentation and biofilm development [44, 45]. Briard *et al*. have demonstrated that phenazines produced by *P*. *aeruginosa* can have stimulatory or antagonistic biological effects on *A*. *fumigatus* depending on its concentrations [15].

Both microscopy and quantification methods enabled us to observe the antibiosis effect of *S*. *maltophilia* on *A*. *fumigatus*. DNA quantification showed a similar growth pattern of *S*. *maltophilia* in the single and mixed biofilms, which was not the case for *A*. *fumigatus* since its filamentation was delayed in the presence of the bacteria after 8 h of co-incubation (Fig 8). The biomass of the mixed biofilm was also much lower between 12 and 24 h than the biomass of the fungal biofilm of the same period (Fig 7). The delay of the biomass and fungal growth appeared between 8 and 12 h, and were still visible until 24 h. The TEM images showed an increase of fungal cell wall thickness in the mixed biofilm that appeared at 12 h and lasted until 16 h. The effect of *S*. *maltophilia* on *A*. *fumigatus* phenotype probably started before 12 h of co-culture. The fungal cell wall thickness increased between 8 and 12 h, then the fungal growth resumed and progressively reached normal growth at 24 h. The antibiosis effect on *A*. *fumigatus* has already been described in the presence of *P*. *aeruginosa* [15] or *S*. *aureus* [35]. Moreover, CLSM showed a difference of the hyphae phenotype between the single fungal and mixed biofilm (Fig 4). The hyphae are highly ramified and the cell wall is thicker in the presence of the bacteria (Fig 5). These findings are entirely opposite to the results of Ramirez Granillo et al. [35] where the fungal structures forming the mixed biofilm of *A*. *fumigatus* and *S*. *aureus* were considerably less dense and filled with many abortive hyphae of small size with lysis appearance. In *A*. *fumigatus—S*. *maltophilia* mixed biofilm, CLSM and TEM observations showed that the hyphae were alive and got adapted to the presence of the bacteria by increasing the thickness of their cell wall (Figs 5 and 6). This adaptation occurred at the beginning of the biofilm formation (between 8 and 12 h), which corresponds to the hyphal formation stage (Fig 6), suggesting that the bacterial molecules responsible for this effect were only active during the germination stage of *A*. *fumigatus*. It has already been demonstrated that bacteria could secrete compounds with anti-fungal effect e.g. maltophilin-like molecules, which have an antifungal activity on *A*. *nidulans* [46, 47]. Many microorganisms can secrete these molecules including *S*. *maltophilia*, which could explain the phenotype observed in *A*. *fumigatus* in our study. Moreover, Li et al. [46, 47] showed that maltophilin irritates *A*. *nidulans* and pushes it to thicken its cell wall which slows down the growth of the fungus. On the other side, Kerr has also shown an inhibitory effect of *S*. *maltophilia* on *A*. *fumigatus* [48]. In the future, studies will be conducted to determine which *S*. *maltophilia* molecules are responsible for the *A*. *fumigatus* cell wall adaptation.

This biofilm model will be used to analyze *in vitro* the interactions between a biofilm and the airway epithelium. For that, we will use primary cultures of pure airways epithelial cells from CF patients [49] to analyze adherence of the biofilm to the airways epithelium and the levels of protein expressions. We might compare the acute phase of inflammatory response between mono-species biofilm and mixed biofilm. It is expected that the response to a mixed biofilm is much higher than the response to a single biofilm. The presence of a mixed biofilm in the airways epithelium, and eventually of an infection by two microorganisms, leads to a more significant chronic inflammation. It also seems important to us to analyze the inflammatory modifications on the whole and the individual roles of fungi and bacteria taking into account the microorganism load in the biofilm [50]. Understanding how this microenvironment operates is likely to provide important insight to the development of effective anti-inflammatory therapies.

In conclusion, using an original biofilm, we demonstrated an antibiosis effect induced by *S*. *maltophilia* on *A*. *fumigatus*. These results need more investigations in order to understand the implication of this interaction *in vivo*, such as in CF patients, where these microorganisms live probably in mixed biofilms. The feasibility of applying this model *in vitro* on the airways epithelium opens the door to analyse the interactions between the bronchial cells and the mixed biofilm, as well as to evaluate the activity of antimicrobial agents on this fungal-bacterial biofilm.
